# Integrated Proteomic and Transcriptomic Investigation of the Acetaminophen Toxicity in Liver Microfluidic Biochip

**DOI:** 10.1371/journal.pone.0021268

**Published:** 2011-08-08

**Authors:** Jean Matthieu Prot, Anne-Sophie Briffaut, Franck Letourneur, Philippe Chafey, Franck Merlier, Yves Grandvalet, Cécile Legallais, Eric Leclerc

**Affiliations:** 1 CNRS UMR 6600, Laboratoire de Biomécanique et Bioingénierie, Université de Technologie de Compiègne, Compiègne, France; 2 CNRS UMR 6599, Heuristique et diagnostique des systèmes complexes, Université de Technologie de Compiègne, Compiègne, France; 3 CNRS UMR 6022, Laboratoire de Génie Enzymatique et Cellulaire, Université de Technologie de Compiègne, Compiègne, France; 4 INSERM U1016, Plateforme de Génomique, Institut Cochin, Paris, France; 5 INSERM U1016, Plateforme de Protéomique, Institut Cochin, Paris, France; Université de Technologie de Compiègne, France

## Abstract

Microfluidic bioartificial organs allow the reproduction of *in vivo*-like properties such as cell culture in a 3D dynamical micro environment. In this work, we established a method and a protocol for performing a toxicogenomic analysis of HepG2/C3A cultivated in a microfluidic biochip. Transcriptomic and proteomic analyses have shown the induction of the NRF2 pathway and the related drug metabolism pathways when the HepG2/C3A cells were cultivated in the biochip. The induction of those pathways in the biochip enhanced the metabolism of the N-acetyl-p-aminophenol drug (acetaminophen-APAP) when compared to Petri cultures. Thus, we observed 50% growth inhibition of cell proliferation at 1 mM in the biochip, which appeared similar to human plasmatic toxic concentrations reported at 2 mM. The metabolic signature of APAP toxicity in the biochip showed similar biomarkers as those reported *in vivo*, such as the calcium homeostasis, lipid metabolism and reorganization of the cytoskeleton, at the transcriptome and proteome levels (which was not the case in Petri dishes). These results demonstrate a specific molecular signature for acetaminophen at transcriptomic and proteomic levels closed to situations found *in vivo*. Interestingly, a common component of the signature of the APAP molecule was identified in Petri and biochip cultures *via* the perturbations of the DNA replication and cell cycle. These findings provide an important insight into the use of microfluidic biochips as new tools in biomarker research in pharmaceutical drug studies and predictive toxicity investigations.

## Introduction

In drug development and toxicity studies, there is increased demand from pharmaceutical companies to develop new approaches that make it possible to focus on optimum drugs at the preclinical stage. The use of toxicogenomics (transcriptomics) has been highlighted to characterize the metabolic consequences of drug toxicity. Cell response at the transcritpome level has contributed to identify new biomarkers and to detect the early symptoms of toxic phenomena *in vitro* and *in vivo* [1]. Recent progress in proteomic analysis has also led to identify biomarkers at the protein level providing new insights into drug development and toxicological science [2], [3]. The combination of the transcriptomic and proteomic approaches contributes to consistently link the biochemical pathways involved in toxicity, and thus improving the descriptions of the toxicological mechanisms of drugs [4].

In order to first clarify and then optimize the therapeutic effects of molecules, new technology platforms, reproducing the targeted tissue of the drugs, represent another new research area. Hepatoxicity is one of the primary causes of late drug withdrawal, mainly because of the lack of a pertinent model for both reproducing functional liver tissue and addressing the systemic toxicity of molecules [5]. Recently, microscale models have been built to cultivate liver cells in order to refine the investigations with hepatocytes [6]. The advantages offered by microfabrication technologies are the design of specific 3D microstructured environments much more elaborate than classical Petri dish cultures [7], [8]. By controlling the microfluidic flow conditions inside these environments, the microscale model now makes it possible to propose successful co-cultures based on various organ cell types (such as liver, lung etc…), reproducing systemic interactions [9]. Highly complex and structured liver microscale models (using human primary hepatocytes and liver non parenchymal cells) can be successfully applied to pharmaceutical drug screening [10], [11].

An important issue for these microscale cultures is the development of dedicated platforms representing alternative methods for *in vivo* screening. Although there is a desire for evidence of the model's performances, there is still a lack of fundamental biological characterizations and comparisons between *in vivo* and *in vitro* data. To determine the real impact of the microscale models, we developed a microfluidic biochip applied to mammalian cell cultures [12], [13]. In the present investigation, we aimed to characterize liver cell responses in the microfluidic biochip using transcriptome and proteome expression profiles. To confirm the advantages of our microfluidic biochip in toxicological and pharmaceutical studies, we compared the effect of a well-known hepatotoxic, the acetaminophen (APAP), on liver cells cultivated in either Petri dishes or in microfluidic biochip culture conditions. APAP toxicity is mainly due to its bioactivation by phase 1 enzyme CYPs into a hypereactive imine: N-acetyl-p-benzoquinone imine (NAPQI) leading to covalent adducts with hepatocyte proteins when GSH cellular stock is depleted. Then, the *in vivo* literature data were compared in order to analyze whether or not the combination of the transcriptomic and proteomic approaches in a microfluidic biochip can improve the understanding of the biochemical consequences of APAP drug toxicity.

## Results

### APAP treatment affects cell morphology, cell cycle repartition and proliferation in microfluidic biochips

**Figure 1:**
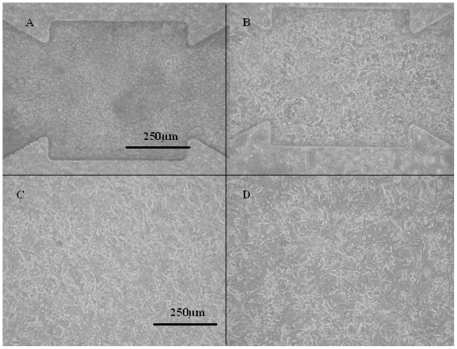
Morphology of the HepG2/C3a cells after 96 hours of culture. (A) biochip without APAP; (B) biochip treated with 1 mM of APAP; (C) Petri dish without APAP; (D) Petri dish treated with 1 mM of APAP.

**Figure 2:**
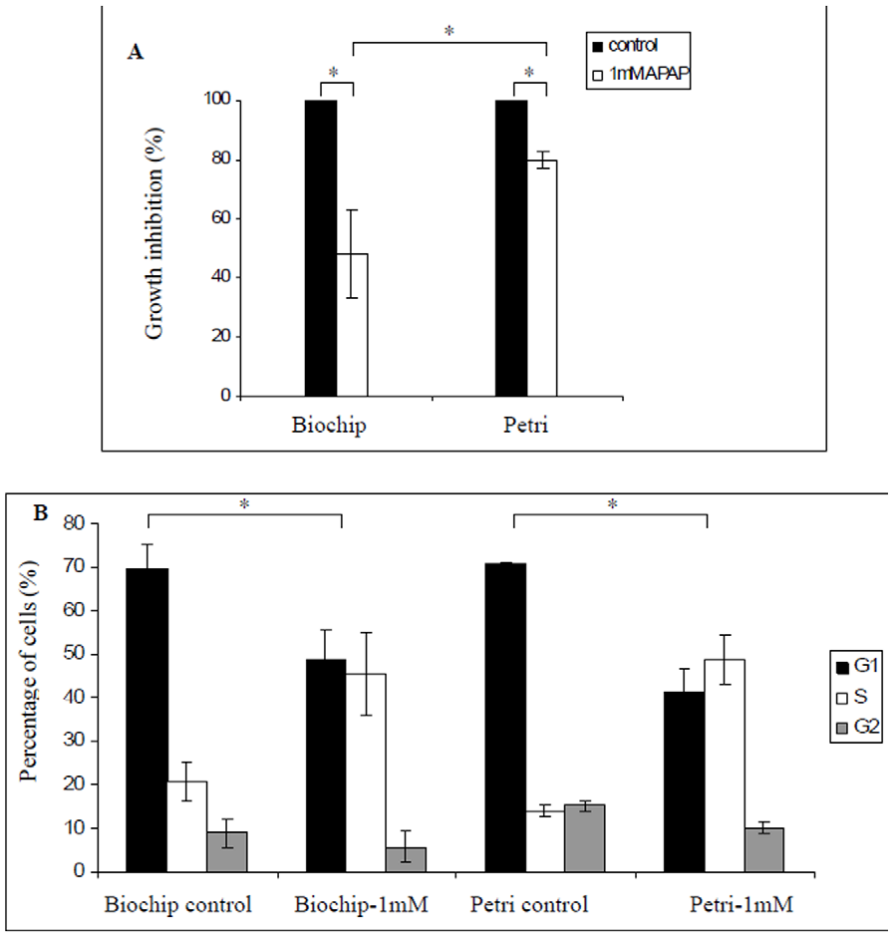
APAP effect on cell proliferation and cell cycle repartition. (A) Comparison of the cell growth in biochip and Petri dishes in untreated and treated conditions with 1 mM of APAP after 96 h of cultures (n = 6, * P<5%); (B) DNA repartition in biochip and Petri dishes after 96 hours of culture. The DNA repartition show for both culture conditions a disruption of the cell cycle repartition compared to control (n = 6* P<5%).

Proliferations of the treated and untreated cells were compared at the end of culture. At 1 mM, we observed the proliferation inhibition of 50% in the microfluidic biochip (Figs. 1A, 1B and Fig. 2A), whereas only 25% of inhibition was found in the Petri dishes (Figs. 1C, 1D and Fig. 2A). In addition, the treatment led to disrupted cell cycle distribution in both conditions, resulting in a blockage in the S phase for both culture systems, as reported in Fig. 2B. In addition, cell apoptosis analysis using flow cytometry (*via* annexin V staining) did not reveal any apoptotic status.

### APAP treatment affects cell metabolism in microfluidic biochips

The metabolic activity was monitored taking albumin secretion and glucose consumption into account as basal cell markers for the functionality of the cells (Table 1). APAP treatment influenced the metabolic activity in both Petri and biochip culture conditions. Compared to the untreated cultures, glucose consumption was measured to be 30% higher in Petri dishes and 37% in biochips in APAP treated conditions. Albumin secretion showed an increase of 40% in both culture conditions

**Table 1:** Basal metabolism (glucose consumption and albumin synthesis), APAP conjugation and CYP1A activity in biochip and in Petri dishes, in treated and untreated cases after 96 h of cultures.

	Glucose consumption(µg/10^6^cell/h)	Albumin synthesis(ng/10^6^cell/h)	Sulfo-APAP(pmol/10^6^cell/h)	Glucurono-APAP	Glutathione-APAP (AU)	CYP1A(pmol/10^6^cell/h)
Biochip control	33±8	88±30	Below LOD	Below LOD	Below LOD	127±20
Biochip treated	51±12	151±49	75±12	Bellow LOD	3	201±36
Petri control	24±3	90±19	Below LOD	Below LOD	Below LOD	Below LOD
Petri treated	34±7	150±46	17±2	Below LOD	Below LOD	Below LOD

Mean ±SD (n>6); LOD = Limit Of Detection (value equal to 100 ng/ml for the sulfo and glucurono-APAP; 20 ng/ml for the Gluthatione APAP, value equal to 80 nmol/L in the EROD assay).

Interestingly, the APAP conjugation activity differed depending on the culture conditions (Table 1). We measured a 3-fold higher secretion of Sulfo-APAP in the biochip when compared to the Petri cultures. In addition, the Gluthatione-APAP production was detected only in the biochip. However, we never detected the Glucurono-conjugation activity, regardless of the culture conditions (Petri or biochip). The production of Glutathione-APAP in the biochip was correlated with significant CYP1A activity, which was maintained only in the biochip, as illustrated by EROD analysis in Table 1.

### Microarray analysis and biologically relevant functions in microfluidic biochips

**Figure 3:**
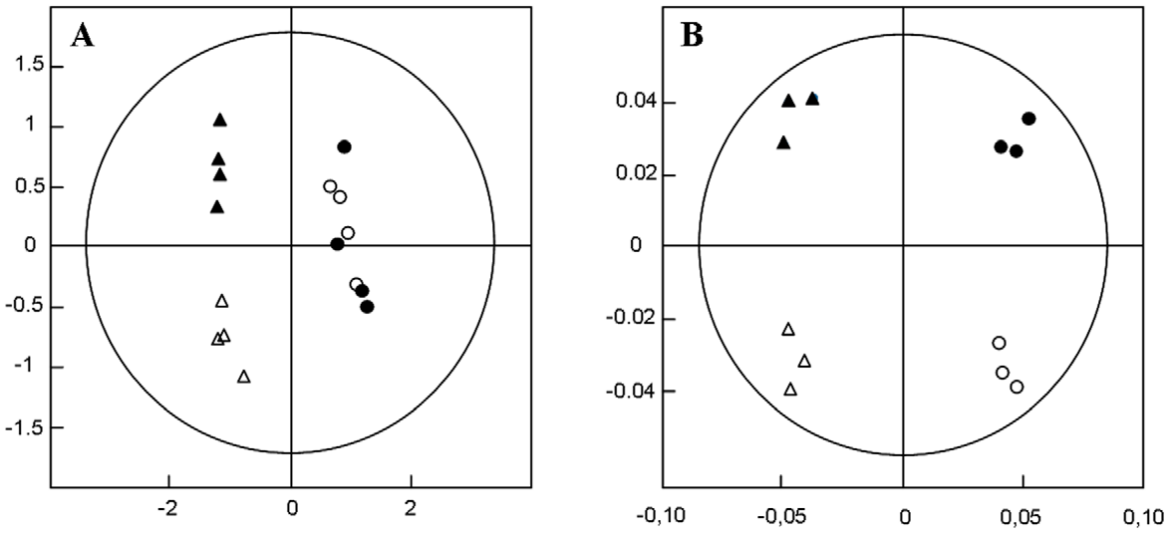
Principal Component Analysis. (A) Proteomic analysis; (B) Transcriptomic analysis; (circles denote Petri data, triangles denote biochips data, black symbols are control data, white symbols are APAP data).

The filtered gene lists led to extract 1236 differentially expressed genes between the Petri dishes and the microfluidic biochip (730 up-regulated and 506 down-regulated by a fold change classification); 144 genes between the untreated biochip and the treated biochip (51 down-regulated and 93 up-regulated, supplement Table S1); and 166 genes between the untreated and treated Petri dishes (23 down-regulated and 143 up-regulated, supplement Table S2). The PCA analysis of the global gene expression in the different conditions is shown in Fig. 3. The first axis of the analysis discriminated the culture conditions by separating two groups: the Petri and the biochip situations. The second axis of the analysis successfully separated the treated and untreated cases. The gene lists were introduced in Ingenuity Pathway Analysis to characterize the biological response of HepG2/C3a cells in relation to the experimental conditions. The software provided the “Top networks”, the “Top canonical pathways”, “Molecular and biological functions associated” and “Top tox lists” (Table 2).

**Table 2:** Ingenuity analysis of the transcriptomic data related to the biochip effect and to the treatment effect.

	Top Networks	Molecular and cellular functions (p value)	Top canonical pathway (p value)	Top tox lists (p value)
**Comparison biochip vs Petri**	1/Drug metab., Lipid metab., Molecular transport	Lipid Metabolism (7×10^−6^)	Biosynthesis of steroids (7×10^−7^)	Cholesterol biosynthesis (3×10^−10^)
	2/ Cellular compromise, Cancer, Cell morphology	Small Molecule biochemistry (7×10^−6^)	Fatty acid metabolism (1×10^−5^)	Fatty acid metabolism (2×10^−5^)
	3/ Cell-mediated Immune response Cellular development, Hematological System Development and Funct.	Cellular growth and proliferation (3×10^−5^)	Butanoate metabolism (3×10^−4^)	LPS/IL1 Mediated inhibition of RXR Function (3×10^−3^)
	4/ Inflammatory response, Gene expression, Cellular movement	Cellular movement (1×10^−4^)	Propanoate metabolism (3×10^−4^)	Oxidative Stress Response Mediated by Nrf2 (1×10^−2^)
	5/Dermatological diseases and conditions, Inflammatory disease, Drug metabolism		Glycerolipid metabolism (5×10^−4^)	PXR/RXR activation (2×10^−2^)
**Treatment effect in biochip**	1/ Cellular development, Haematological system development and Function, Cellular development	Cell morphology (2×10^−4^)	D-arginine and D-ornithine Metabolism (8×10^−3^)	VDR/RXR activation (2×10^−2^)
	2/ Gene expression, Cell death, Cell cycle	Cellular movement (3×10^−4^)	Sphingolipid metabolism (1×10^−2^)	CYP450 panel (5×10^−2^)
	3/ Cancer, Cell cycle, Gene expression	DNA replication, recombination and repair (2×10^−3^)	VDR/RXR activation (2×10^−2^)	Hormone receptor regulated cholesterol metabolism (5×10^−2^)
	4/Cell-To-Cell Signalling and Interaction, Immune Cell Trafficking	Cell cycle (2×10^−3^)	Glycosaminoglycan degradation (2×10^−2^)	
	5/Cell Signalling,Embryonic Development,Tissue Development	Lipid metabolism (2×10^−3^)	Pyrimidine metabolism (8×10^−2^)	
**Treatment effect in Petri**	1/ DNA Replication Recombination and Repair	Cell cycle (9×10^−19^)	Role of BRCA1 in DNA Damage Response (4×10^−10^)	p53 signalling (4×10^−8^)
	2/ Cell Cycle, Cancer, Reproductive System Disease	DNA replication, recombination and repair (1×10^−9^)	Role of CHK Proteins in Cell Cycle Checkpt Ctrl (1×10^−9^)	G2/M transition of the cell cycle (5×10^−6^)
	3/Cell cycle, Cancer Cell Morphology	Cellular assembly and organisation (6×10^7^)	p53 Signalling (4×10^−8^)	G1/S transition of the cell cycle (4×10^−4^)
	4/Cell Death, Cancer, Reproductive System Disease	Cellular growth and proliferation (3×10^−6^)	Mitotic Roles of Polo-Like Kinase (1×10^−7^)	AHR signalling (8×10^−5^)
		Cell compromise (1×10^−5^)	Pyrimidine Metabolism (1×10^−6^)	

**Figure 4:**
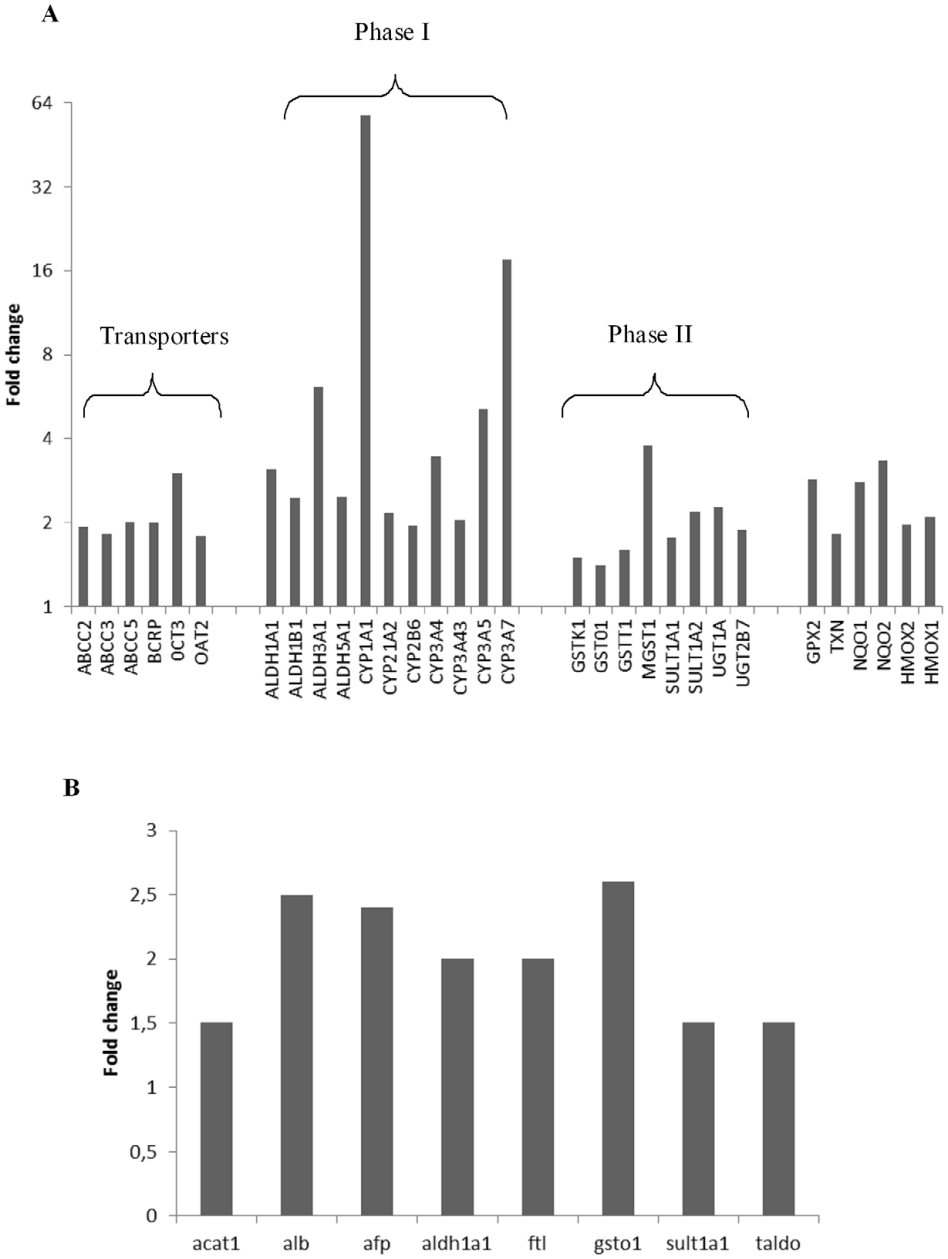
Mean genes and proteins affected by the culture condition. (A) Mean genes differentially expressed by the environment condition and involved in hepatic differentiated function; (B) Mean proteins differentially expressed by the environment condition and involved in hepatic differentiated function.

The comparison between the biochip and Petri (without APAP treatment) showed that “drug and lipid metabolisms” and “molecular transport” appeared in the “Top networks” significantly affected by the microfluidic culture conditions. Since HepG2/C3a cells are clones derived from a hepatocarcinoma, “cell compromise” and “cancer pathway” were also extracted. Then, in the “ToxList” we found the RXR/PXR activation pathway (involved in drug metabolism) and an inflammatory response via the NRF2 pathway. The genes involved in xenobiotic metabolism (phase 1 and phase 2 enzymes, phase 3 transporters) were particularly over expressed in the biochip, as shown in Fig. 4A.

In the biochips, the perturbations triggered by APAP are presented in Table 2. Among the pathways, “VDR/RXR activation” appeared to be affected. This pathway is involved in the hepatic bioconversion of vitamin D and regulation of Ca^2+^ homeostasis [14]. “Cell morphology” and lipid metabolism were also affected when compared to the treated Petri.

In the Petri dishes, different pathways, when compared to the treated biochips, were specifically highlighted such as “DNA damage response”, “cell cycle checkpoint control”, “p53 signaling” and the “BRCA1 response” (Table 2). These pathways appeared in apoptosis and cell proliferation regulation.

Finally, 36 genes were commonly affected by the treatment regardless of the culture situations (in Petri dishes or in the biochip). These commons genes were involved in “cell cycle”, “DNA replication recombination” and “repair functions”. This result appeared consistent with the cell cycle repartition analysis presented in Fig. 2B. “Pyrimidine metabolism” appeared to be equally affected by the treatment regardless of the culture situation.

### Proteomic analysis and biological relevant functions in microfluidic biochips

The proteomic analysis highlighted 111 candidate proteins differentially expressed when we compared the culture conditions and treatments. The PCA reported in Fig. 3 successfully distinguished the culture condition between the Petri dishes and biochip whereas the effect of the treatment was only identified in the biochip culture conditions.

We found 86 proteins that were differentially expressed between the Petri dishes and biochip without treatment (40 up-regulated and 46 down-regulated, supplement Table S3). Of the specific hepatic markers, we found that the protein expression of albumin (ALB), aldhedhyde dehydrogenase (ALDH1A1), and GSTO1 were overexpressed (Fig. 4B).

The treatment in the biochip contributed to identify 27 protein spots differentially expressed when compared to the untreated biochip (17 up-regulated and 10 down-regulated, supplement Table S4). The proteomic analysis showed that cell response in the biochip due to the APAP induced a modification in the cell process related to the “calcium binding and homeostasis” (*via* annexin A7 and visinin) and related to lipid binding, proteolysis and cytosqueleton (*via* Coronin, Actin, Keratine, Tubulin). Other specific markers detected in biochips were involved in cell cycle regulation process (PP2CA, Stratifin), the apoptosis cascade (Caspase 3, Cathepsin B), glucose metabolism (G6PD) and pyrimidine metabolism (Thioredoxin 1).

In the Petri dishes, we found only 8 protein spots differentially expressed resulting from the APAP (6 up-regulated and 2 down-regulated, supplement Table S5). Although the PCA did not distinguish the treatment effect in the Petri dishes, the identified proteins from the Coomassie gel were involved in DNA replication and the cell cycle regulation process (MCM7, RBBP4, PCNA and Nucleolin), as well as cytoskeleton organization (CAP2).

Finally, three identified proteins showed a common alteration between the Petri dishes and biochip conditions because of the APAP treatment: Annexin A7, Coronin 1B, and the CK8.

## Discussion

### Transcriptomic and Proteomic crosslinks in the microenvironment

The integration of the transcriptomic and proteomic analyses revealed a significant correlation with the environment effect. Totally, 26 protein markers detected in the proteomic analysis also appeared at the gene levels. Ingenuity pathway analysis of the resulting combination markers for genes and proteins highlighted the NRF2 pathway and fatty acid metabolism. This NRF2 pathway is related to oxidative stress and xenobiotic response. In biochip, it led to the over-expression of genes and proteins involved in glutathione metabolism, protein ubiquitination, phase 1 (CYP P450) and phase 2 (SULT, GST, UGT) enzymes and phase 3 transporters (ABCC). This stimulated pathway was the result of the microenvironment and microfluidic culture conditions without the APAP loading. Despite this cytoprotective response in the microfluidic biochip, no apoptotic situation was attained. Stimulation of the ARE (the anti-oxidant responsible element) by the NRF2 transcription activator resulted in over-expression of most of the genes found in the transcriptomic analysis including: VCP, HSP90, STIP1 in the removal and reparation of damaged proteins; CYPs450, GST, NQO, UGT, SULT, EPHX1, GCLM, CBR1, and AKR as phase 1 and phase 2 enzymes; SQTSM1, HO-1, PRDX1, FTL, FTH1, CAT, GPX2, SOD, TXN, and GSR as antioxidant proteins. In addition, this induction was clearly confirmed at the proteome level. At the protein level, we found the over-expression of the specific protein ALDH1A1, FTL (ferritin), G6PD, GSTO1, HSP90, SULT1A, and they were also involved in the pathway [15].

### Towards predictive toxicity in microfluidic biochips

The NRF2 pathway is an early inflammatory response that induced the genes involved in the detoxication process and the response related to oxidative stress. This early stress was attributed to early cell adaptation to the micro environment [16]. In our case, NRF2 pathway activation led to a more sensitive response to APAP treatment when compared to Petri conditions. APAP overdose is the most common form of drug poisoning in the USA, resulting in acute liver failure caused by a necrosis of the hepatocytes in the centrilobular region. The initial offstage in toxicity was highlighted by Gillette's works and is defined by the formation of a hyperactive quinone-imine, NAPQI, via the bioactivation of APAP by CYPs450. At therapeutic doses (less than 4 g per day), APAP is conjugated via UDP glucuronotransferase or sulfotransferase whereas at higher concentrations this detoxication process is saturated. NAPQI is trapped by Glutathione via the GST enzyme until depletion of the glutathione stock, leading to covalent binding to the protein [17]–[19]. Classical studies demonstrating the hepatotoxic effect of acetaminophen performed *in vitro* show that cytotoxicity is observed for a concentration of APAP ranging up to 5 to 20 mM with interspecies differences [20]. In our case, acetaminophen led to an EC50 at a 1 mM concentration for 72 hours of contact only in the microfluidic biochip configuration. This result is in accordance with the toxic plasmatic level observed in humans and which ranges between 1 and 2 mM [21], [22]. However, we did not take into account the protein binding to acetaminophen. Despite acetaminophen has a weak affinity for plasma proteins (<20% [23], [24]), the protein contents between blood and culture are different. Thus, the protein binding quantification will be necessary to fully confirm that the microfluidic biochip condition is a more “physiological-like” *in vivo* situation.

Furthermore, several studies have shown that acetaminophen could play a critical role in the cell proliferation process [25], [26]. Interestingly, this effect results also in a perturbation of the DNA replication in a CYP independent manner with blockage of the transition of G0/G1 or S phases. This result also explained why we found in both biochips and Petri configurations the similar effect on cell cycle progression in presence or absence of bio activation of APAP. However, we detected the APAP-GSH conjugate only in the biochips, illustrating that acetaminophen is bioactivated into NAPQI in this configuration. This observation was correlated with the significant CYP1A activity observed in the biochips. CYP1A is known to bioactivate acetaminophen as demonstrated *in vivo* in the double null CYP1A2/CYP2E1 mice [27] in which mice were protected from APAP toxicity through the absence of these enzymes. APAP might thus be biotransformed via the CYP1A in the biochips and produce the hepatotoxic compound NAPQI. In the biochips, the NAPQI is then trapped by the glutathione stock and excreted by the cells in its inactive form as demonstrated by the apparition of Glutathione-APAP. The depletion of the glutathione cell stock led to covalent binding of NAPQI to cell components possibly leading to cell perturbation until cell death. This mechanism may explain the higher cell growth inhibition observed in the biochips when compared to the Petri dishes.

### Toxicogenomics in the microfluidic biochip highlights specific and physiological APAP biomarkers

144 genes and 27 proteins were altered when cells were treated in microfluidic biochips. Among them, only four markers appeared to be affected at the transcriptomic and proteomic levels. At the gene level, the hepatic bioconversion of vitamin D and regulation of Ca^2+^ homeostasis [14] (via VDR/RXR with HES1, HSD17B2, and NCOA1 genes) were affected in the microfluidic biochip. At the proteome level, we found a down-regulation of proteins involved in protein synthesis (such as HNRNPC1/C2, Serine-threonine kinase receptor-associated protein), and of the proteins involved in the calcium ion binding (such as Annexin A7, Visinin-like protein 1 and S100P). An increase in cytosolic calcium concentration is a common death signal resulting from acetaminophen treatment [28]. This phenomenon was explained by the arylation (inhibition) of the calcium pump (Ca^2+^-ATPase) by NAPQI, leading to an accumulation of cytosolic calcium [28]–[30]. Furthermore, the loss of mitochondria permeability induced by NAPQI binding (the mitochondria being involved in the Ca^2+^ sequestration) also led to an increase in cytosolic Ca^2+^ accumulation [28]–[30]. This increase was reported to be enough to activate nucleolar endonucleases and proteases such as calpain in *in vivo* mice studies resulting from APAP toxicity and leading to cell injury [31], [32].

Moreover, based on a metabonomic approach, it has been shown that liver APAP hepatotoxicity induced elevated lipid concentrations related to lipid metabolism failure in mouse livers [33]. This APAP hepatoxicity was also correlated with over-expression of the insulin-like growth factor binding protein-1 gene (IGFBP-1). Interestingly, we found common results in the biochip insomuch as at the transcriptional level the APAP induced down-regulation of lipid metabolism genes (such as GPD1, GPAM, DAK) and up-regulation of IGFBP-1 (which was not the case in the Petri dishes). In addition, at the proteome level, we found that some enzymes involved in lipid production (such as leukotriene A-4 hydrolase, short-branched chain specific acyl-CoA dehydrogenase, and hydroxymethylglutaryl-CoA synthase) were still up-regulated in the biochip when compared to Petri cultures.

Finally, we also found a decrease in actin, tubulin and coronin-1b synthesis at the protein levels involved in cytoskeleton reorganization in the microfluidic biochip. This was correlated at the gene levels *via* the modification in the genes group “Cell morphology” of the “cell and molecular function” category of the Ingenuity analysis (such as TIMP1, TIMP2, RACGAP1, BTG2, UCN2, RRAS, KIF14 and CBX5, DNMT3B, LGMN, MCAM, RRAS). Interestingly, cytoskeleton reorganization has also been reported in the literature as an *in vivo* APAP toxicity consequence [34]. The combination of the genes and proteins affected in the microfluidic biochip has thus played a part in the identification of a specific biomarkers signature that appeared closer to the *in vivo* one when compared to Petri dishes.

In conclusion, we have analyzed the transcriptomic and proteomic profiles of HepG2/C3a cultivated in microfluidic biochips and Petri dishes. The profiles integration have shown an induction of a cytoprotective response in the biochip leading to the induction of the cell defense mechanisms. When applied to acetaminophen toxicity analysis, the microfluidic biochip cultures have demonstrated a higher physiological response when compared to Petri cultures. In Petri and biochip conditions, the cell cycle and cellular reorganization were modified by the APAP. However, the limitation of the HepG2/C3a proliferation was more important in the biochip leading to reach an IC_50_ at 1 mM of APAP (IC_25_ in Petri respectively). This was attributed to a probable NAPQI synthesis in biochips due to the induction of the drug metabolism related genes in the micro environment. Among the metabolic network affected by the APAP in biochip, we found the lipid metabolism and the VDR/RXR pathway related to the calcium homeostasis. Finally, our finding provides a new insight into the use of microfluidic biochips as new tools in toxicity and mechanistic researches in pharmaceutical drug studies and predictive toxicity investigations

## Materials and Methods

### Microfluidic biochip fabrication

**Figure 5:**
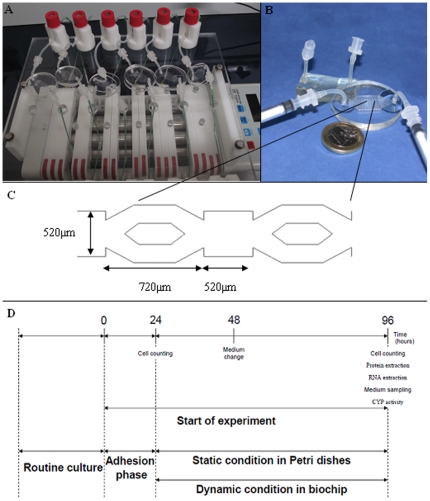
Experimental design description. (A) Perfusion setup and peristaltic pump setup with 6 individual biochips and medium reservoirs; (B) Microfluidic biochip; (C) Microchannel design inside of the biochip; (D) Experimental procedure.

The fabrication details, based on replica molding of polydimethylsiloxane (PDMS) and the choice of microfluidic biochip design, have been described previously [12], [13]. In summary, the biochip's construction includes a first PDMS layer with the microstructures for cell attachment, which contains a series of microchambers and microchannels inside a larger cell culture chamber. The biochip has a volume estimated at 40 µL (Fig. 5).

### Cell culture conditions

HepG2/C3a was obtained from the American Type Culture Collection (ATCC, reference CRL 10-741) and cultivated according the ATCC recommendations. Stock solution of acetaminophen (APAP) was prepared by direct dilution of powder (SIGMA) in the medium before dilution at 1 mM. Cell culture conditions were previously described. Briefly cells were seeded in biochips and in 12-well culture plates (Becton Dickinson), at the same density of 0.25×10^6^ cells/cm^2^. Dynamic condition in biochip was performed after 24 h of adhesion at 10 µl/min. The APAP was loaded in the biochip circuit and Petri dishes before to start the perfusion.

In dynamic flow experiments, the biochip were introduced into a 3 mL circuit loop through the use of a peristaltic pump and a bubble trap interconnected by silicone tubing with a 1.0 mm interior diameter. The flow rates were controlled by the pump and the bubble trap was used as a 2 ml culture medium reservoir. The cultures were performed simultaneously in non-treated and treated APAP conditions.

### Cell cycle analysis and apoptosis evaluation

Cells were trypsinized inside the biochip, collected and washed with 1 mL of PBS containing 5 mM EDTA. They were then fixed for 45 min at 4°C in 1 mL of 75% ethanol in PBS with 5 mM EDTA, washed, suspended in PBS containing 5 mM EDTA, 0.1% Triton X-100 (Promega), 40 µg RNase A (Sigma) and 25 µg propidium iodide (PI, Sigma), and incubated for 15 min protected from light. The stained samples were analyzed in an Epics XL-MCL flow cytometer (Beckman Coulter). The histograms were analyzed using Wincycle software.

Cell apoptosis was also evaluated by the quantification by cytometer (Epics XL-MCL flow cytometer (Beckman Coulter) of the annexin V positive cells. The cells were detached by trypsinization, washed with PBS and resuspended in binding buffer (10 mM Hepes/NaOH, pH 7.5 containing 0.14 M NaCl and 2.5 mM CaCl_2_). Cell suspension was stained with 0.25 µg of annexinV/FITC and 10 µg of Propidium Iodide and analysed.

### Metabolism activities

Glucose consumption and albumin production were measured after 96 h of culture. The protocols have been described in detail in our previous works [12]. Briefly, the glucose was measured using a Konelab 20 biochemical analyzer (Thermo Electron Corporation). Albumin synthesis was measured by means of an ELISA sandwich technique (anti Human Albumin IgG, Cappel; anti Human Albumin IgG coupled with peroxydase, Cappel).

CYP1A1/2 activities were determined using 5-ethoxyresorufin (10 µM) as substrate. Resorufin formation by 7-ethoxyresorufin O-deethylation (EROD) was quantified with a fluorescence microplate reader (TECAN, Spectrafluor plus) after 1 hour's incubation in the presence of salicylamide (3 mM) in order to inhibit phase II enzymes. Fluorescence was determined at λ_ex_ 535 nm and λ_em_ 595 nm.

APAP metabolites were measured using the LC/MS/MS method. Medium samples containing APAP were collected at the end of the culture period. An aliquot of 10 µL of culture medium was diluted 3 times in A before LC-MS/MS analysis. The LC-MS/MS system is composed of Dionex Ultimate 3000 capillary HPLC with a Famos injector and a UV UVD 3000 detector. The HPLC chain is coupled with a Triple Quad WATERS (micromass) Quatro micro mass spectrometer. The separation process was performed using a Thermo Hypercyl Gold Aq (150×0.5 mm×3 µm) colonna. The mobile phase is constituted with A (Acetate ammonium 10 mM in water) and B (Acetonitrile 50% and 50% of A) with a flow rate of 8 µl/min. The gradient of the mobile phases consists of 5% B in A at 0 min followed by a linear gradient with 43% of B at 20 min until 100% of B at 40 min and back to 5% of B at 41 min. The analytes were detected by MRM (Multiple Reaction Monitoring) in positive ion mode. The different areas obtained for Glutathione-APAP (MW: 457 g/mol), Glucurono-APAP (MW: 328 g/mol), and Sulfo-APAP (MW: 232 g/mol) were compared to a known quantity of each standard (SIGMA) making possible a semi-quantitative dosage.

### RNA extraction, hybridization on Affymetrix chips and microarray analysis

For the microarray study, the cells were trypsined from the biochips and Petri dishes in triplicate. Total RNA extraction was performed after 96 h of culture (Fig. 5). The total RNA was extracted using a Nucleospin® RNA XS isolation kit (Macherey-Nagel). The quantity of RNA was assessed with a Nanodrop ND-1000 spectrophotometer (Nyxor Biotech, Paris, France). RNAs quality was verified with a Bioanalyzer 2100 (Agilent Technologies, Massy, France). RIN ranged between 9.3 and 10.

The raw data (affymetrix .cel files) were obtained using affymetrix Genechip operating software. All .cel files were analyzed using the affymetrix expression console in order to monitor microarray quality with different control metrics. Data were normalized using Robust Multichip Average methodology (RMA) in order to remove handling errors. A PCA (Principal Component Analysis) was applied to the data using R program. Mean expression values of the triplicate were calculated and the gene lists were filtered according to the fold change (on contrary to our previous work [16]) in order to conserve only the genes with a fold change of more than 1.8 (up-regulated) or less than 0.55 (down-regulated). Lists of the genes corresponding to the different experimental conditions were introduced into the ingenuity pathway to obtain biological functions, top network and gene ID.

### Proteomic analysis: 2D Dige

The method follows the instructions of the constructor (Ettan Dige User manual, GE Healthcare) and the experimental design is represented in supplement table S6. Briefly, the cells were collected and the protein concentration determined by a Bradford method. Then, the proteins were labelled with CyDye DIGE fluor kit. Labelled samples were mixed in the rehydratation buffer before to be loaded onto the 18-cm IPG strips (GE-Healthcare) with equal amount (50 µg). After passive rehydratation, isoelectrofocalisation of the different strips was performed using an IPGphor apparatus (GE Healthcare) for a total of 70 kV.h. At the end of the cycle, strips were stocked at −20°C before equilibration step. Equilibrated strips were placed onto homemade polyacrylamide gels (8–18%), overlaid with agarose solution and electrophoresis was performed simultaneously in a Ettan-DALT II system (GE Healthcare) at 2.5 W/gel at 15°C until the bromophenol blue dye reached the bottom of the gels. Gels were scanned using a Typhoon 9400 (GE Healthcare) with a resolution set at 100 µm. Image analysis were performed by Decyder software suite (GE Healthcare, version 5.02) which allow the comparison of the different combination corresponding to the experimental conditions. Down or up-expressed proteins of the different experimental conditions (microfluidic biochip, petri dishes) were retained if protein spot fold change was larger than +1.5 or smaller than −1.5 and a Student's t-test p-value less than 0.05. PCA (Principal Component Analysis) was performed on the global proteins distribution to see the different repartition according the experimental conditions. Spots of interest were manually excised on Coommassie Blue gel by visual comparison with 2D-Dige master gel. For MS and MS/MS analysis, the sample analysis was performed using a MALDI-TOF-TOF 4800 mass spectrometer (Applied Biosystems). Spectra acquisition and processing was performed using the 4000 series explorer software (ABI) version 3.5.28193 in positive reflectron mode at fixed laser fluency with low mass gate and delayed extraction. Database searching was carried out using Mascot version 2.2 (MatrixScience, London, UK) via GPS explorer software (ABI) version 3.6 combining MS and MS/MS interrogations on Human proteins from Swissprot databank, 18138 entries, (Swissprot databank: 333445 sequences; 120048673 residues, www.expasy.org). The search parameters were as follows: carbamidomethylation as a variable modification for cysteins and oxidation as a variable modification for methionines. Up to 1 missed tryptic cleavage was permitted and mass accuracy tolerance of 30 ppm for precursors and 0.3 Da for fragments were used for all tryptic mass searches. Positive identification was based on a Mascot score above the significance level (i.e. <5%). The reported proteins were always those with the highest number of peptide matches. Under our identification criteria, no result was found to match to multiple members of a protein family.

## Supporting Information

Table S1 Differentially expressed genes by the APAP treatment in biochip when compared to the untreated biochip (fold change above 1.8 or below 0.55).
(DOC)

Table S2 Differentially expressed genes by the APAP treatment in Petri when compared to the untreated Petri (fold change above 1.8).
(DOC)

Table S3 Proteins differentially expressed by the biochip versus the Petri and successfully identified by MS/MS.
(DOC)

Table S4 Differentially expressed proteins by the treatment effect in biochip and successfully identified by MS/MS.
(DOC)

Table S5 Differentially expressed proteins by the treatment effect in Petri dishes and successfully identified by MS/MS.
(DOC)

Table S6 Experimental design and samples labelling of the proteomic analysis. Each biochip and Petri is the pool of three independent replicates.
(DOC)
