# Supplementary material for: Integrated Proteomic and Transcriptomic Investigation of the Acetaminophen Toxicity in Liver Microfluidic Biochip
Source: PLoS One. 2011 Aug 8;6(8):e21268. doi: 10.1371/journal.pone.0021268 (PMC3152546; doi:10.1371/journal.pone.0021268)
Supplement: Table S1 — Differentially expressed genes by the APAP treatment in biochip when compared to the untreated biochip (fold change above 1.8 or below 0.55). (DOC) [file pone.0021268.s001.doc]

**Supplementary table 1:** Differentially expressed genes by the APAP treatment in biochip when compared to the untreated biochip (fold change above 1.8 or below 0.55).

| **Affymetrix** | **Gene Name** | **Complete Name** | **Fold Change** |
| --- | --- | --- | --- |
| 8018038 | ABCA5 | ATP-binding cassette, sub-family A (ABC1), member 5 | 1.84 |
| 8049737 | AGXT | alanine-glyoxylate aminotransferase | 0.51 |
| 7934979 | ANKRD1 | ankyrin repeat domain 1 (cardiac muscle) | 2.8 |
| 8073062 | APOBEC3B | apolipoprotein B mRNA editing enzyme, catalytic polypeptide-like 3B | 2.12 |
| 7944035 | APOC3 | apolipoprotein C-III | 0.47 |
| 7982287 | ARHGAP11B | Rho GTPase activating protein 11B | 1.83 |
| 8171392 | ASB9 | ankyrin repeat and SOCS box-containing 9 | 0.54 |
| 8034772 | ASF1B | ASF1 anti-silencing function 1 homolog B (S. cerevisiae) | 1.87 |
| 7915592 | other | RNA, U5F small nuclear | 0.52 |
| 7972979 | other | similar to C21orf99 protein | 0.39 |
| 8124926 | BAT1 | HLA-B associated transcript 1 | 0.52 |
| 8170648 | BGN | biglycan | 2.49 |
| 8015769 | BRCA1 | breast cancer 1, early onset | 1.83 |
| 8017262 | BRIP1 | BRCA1 interacting protein C-terminal helicase 1 | 1.86 |
| 7969374 | C13orf34 | chromosome 13 open reading frame 34 | 1.92 |
| 7985873 | C15orf42 | chromosome 15 open reading frame 42 | 1.95 |
| 8115397 | C5orf4 | chromosome 5 open reading frame 4 | 2.02 |
| 8178439 | C6orf15 | chromosome 6 open reading frame 15 | 0.4 |
| 8119918 | C6orf223 | chromosome 6 open reading frame 223 | 2 |
| 7899346 | CCDC72 | coiled-coil domain containing 72 | 0.5 |
| 8145418 | CDCA2 | cell division cycle associated 2 | 1.8 |
| 8168794 | CENPI | centromere protein I | 2.06 |
| 7929334 | CEP55 | centrosomal protein 55kDa | 1.97 |
| 7985213 | CHRNA5 | cholinergic receptor, nicotinic, alpha 5 | 1.83 |
| 8054702 | CKAP2L | cytoskeleton associated protein 2-like | 2.27 |
| 7926896 | CKS1B///LOC652904 |  | 1.94 |
| 8035517 | COMP | cartilage oligomeric matrix protein | 1.89 |
| 7971444 | CPB2 | carboxypeptidase B2 (plasma) | 0.4 |
| 7951662 | CRYAB | crystallin, alpha B | 2.04 |
| 8162652 | CTSL2 | cathepsin L2 | 2.67 |
| 8018754 | CYGB | cytoglobin | 2 |
| 8067140 | CYP24A1 | cytochrome P450, family 24, subfamily A, polypeptide 1 | 3.44 |
| 8134655 | CYP3A43 | cytochrome P450, family 3, subfamily A, polypeptide 43 | 2.72 |
| 8026456 | CYP4F3 | cytochrome P450, family 4, subfamily F, polypeptide 3 | 1.89 |
| 8111772 | DAB2 | disabled homolog 2, mitogen-responsive phosphoprotein (Drosophila) | 0.52 |
| 7940451 | DAK | dihydroxyacetone kinase 2 homolog (S. cerevisiae) | 0.54 |
| 7956046 | DGKA | diacylglycerol kinase, alpha 80kDa | 2.3 |
| 7905025 | DRD5 | Dopamine receptor D5 | 2.16 |
| 7965094 | E2F7 | E2F transcription factor 7 | 2.18 |
| 8008310 | EME1 | essential meiotic endonuclease 1 homolog 1 | 1.88 |
| 7924619 | ENAH | enabled homolog (Drosophila) | 2 |
| 8145570 | ESCO2 | establishment of cohesion 1 homolog 2 (S. cerevisiae) | 1.94 |
| 7940147 | FAM111B | family with sequence similarity 111, member B | 1.81 |
| 8136115 | FAM40B | family with sequence similarity 40, member B | 1.82 |
| 8022803 | FAM59A | family with sequence similarity 59, member A | 1.82 |
| 7904452 | FAM72A |  | 1.88 |
| 8108205 | FBXL21 | F-box and leucine-rich repeat protein 21 | 2.7 |
| 8152031 | FBXO43 | F-box protein 43 | 1.85 |
| 8105302 | FST | follistatin | 2.18 |
| 7986092 | FURIN | furin (paired basic amino acid cleaving enzyme) | 2.39 |
| 7981752 | GOLGA8E | Golgi autoantigen 8E | 0.46 |
| 8165663 | GPAM | Glycerol 3 phosphate acetyl transferase 1 | 0.51 |
| 7955348 | GPD1 | glycerol-3-phosphate dehydrogenase 1 (soluble) | 0.49 |
| 7996081 | GPR56 | G protein-coupled receptor 56 | 2.02 |
| 8027819 | HAMP | hepcidin antimicrobial peptide | 1.97 |
| 7997188 | HP |  | 0.41 |
| 7997491 | HSD17B2 | hydroxysteroid (17-beta) dehydrogenase 2 | 1.94 |
| 8132694 | IGFBP1 | insulin-like growth factor binding protein 1 | 1.84 |
| 7981722 | IGHA1///IGHG1 | immunoglobulin heavy constant alpha 1 | 0.37 |
| 7921033 | IQGAP3 | IQ motif containing GTPase activating protein 3 | 1.89 |
| 7947248 | KIF18A | kinesin family member 18A | 2 |
| 8092348 | LAMP3 | lysosomal-associated membrane protein 3 | 1.94 |
| 8165298 | LCN15 | lipocalin 15 | 2.6 |
| 8092541 | LIPH | lipase, member H | 2.34 |
| 7981728 | LOC100290036 | Protein coding | 3.82 |
| 7998405 | LOC146336 | hypothetical protein FLJ32252 | 0.53 |
| 8113709 | LOX | lysyl oxidase | 0.52 |
| 7935553 | LOXL4 | lysyl oxidase-like 4 | 2.26 |
| 7907893 | MR1 | major histocompatibility complex, class I-related | 2.16 |
| 8090180 | MUC13 | mucin 13, cell surface associated | 1.86 |
| 8151101 | MYBL1 | v-myb myeloblastosis viral oncogene homolog (avian)-like 1 | 1.89 |
| 7922773 | NCF2 | neutrophil cytosolic factor 2 | 1.97 |
| 8098423 | NEIL3 | nei endonuclease VIII-like 3 (E. coli) | 2.36 |
| 7924096 | NEK2 | NIMA (never in mitosis gene a)-related kinase 2 | 1.95 |
| 7957835 | NR1H4 | nuclear receptor subfamily 1, group H, member 4 | 0.51 |
| 8095456 | ODAM | odontogenic, ameloblast asssociated | 0.5 |
| 8117622 | OR2B6 | olfactory receptor, family 2, subfamily B, member 6 | 2.6 |
| 7911283 | OR2T3///OR2T34 | Olfactory receptor, family 2, subfamily T, member 3 | 0.53 |
| 8066619 | PLTP | phospholipid transfer protein | 2.39 |
| 8119858 | POLH | polymerase (DNA directed), eta | 1.89 |
| 8089875 | POLQ | polymerase (DNA directed), theta | 1.91 |
| 8118509 | PPT2///EGFL8 | other | 2.08 |
| 8052418 | PUS10 | pseudouridylate synthase 10 | 0.54 |
| 7916112 | RAB3B | RAB3B, member RAS oncogene family | 1.87 |
| 7908125 | RGL1 | Ral guanine nucleotide dissociation stimulator-like 1 | 1.84 |
| 7948894 | RNU2-2///RNU2-1///WDR74 | small nucleolar RNA, C/D box 22 | 0.46 |
| 8001918 | RRAD | Ras-related associated with diabetes | 1.8 |
| 8038407 | RRAS | related RAS viral (r-ras) oncogene homolog | 1.81 |
| 7920278 | S100A3 | S100 calcium binding protein A3 | 1.93 |
| 7943158 | SCARNA9 | small Cajal body-specific RNA 9 | 1.9 |
| 7943160 | SCARNA9 | small Cajal body-specific RNA 9 | 1.86 |
| 8008646 | SCPEP1 | serine carboxypeptidase 1 | 1.87 |
| 8135069 | SERPINE1 | serpin peptidase inhibitor, clade E (nexin, plasminogen activator inhibitor type 1), member 1 | 1.83 |
| 7944867 | SIAE | musculus sialic acid acetylesterase | 0.51 |
| 8124365 | SLC17A2 | solute carrier family 17 (sodium phosphate), member 2 | 0.54 |
| 8064613 | SLC4A11 | solute carrier family 4, sodium borate transporter, member 11 | 2.43 |
| 7960177 | SLC6A12 | solute carrier family 6 (neurotransmitter transporter, betaine/GABA), member 12 | 2.1 |
| 8112469 | SMA4 | SMA4 | 0.54 |
| 7982058 | SNORD115-1 | small nucleolar RNA, C/D box 115-1 | 0.52 |
| 7982046 | SNORD115-3 | small nucleolar RNA, C/D box 115-3 | 0.49 |
| 7948898 | SNORD31///SNHG1 | small nucleolar RNA, C/D box 31 | 0.55 |
| 8104035 | SORBS2 | sorbin and SH3 domain containing 2 | 0.55 |
| 7994603 | SPN | sialophorin | 2.01 |
| 8060997 | SPTLC3 | serine palmitoyltransferase, long chain base subunit 3 | 0.54 |
| 8168749 | SRPX2 | sushi-repeat-containing protein, X-linked 2 | 1.85 |
| 8057887 | STK17B | serine/threonine kinase 17b | 1.88 |
| 8163896 | STOM | stomatin | 1.9 |
| 8158059 | STXBP1 | syntaxin binding protein 1 | 1.86 |
| 8066822 | SULF2 | sulfatase 2 | 2.15 |
| 8108217 | TGFBI | transforming growth factor, beta-induced, 68kDa | 1.83 |
| 8167185 | TIMP1 | TIMP metallopeptidase inhibitor 1 | 2.01 |
| 7964347 | TMEM194A | transmembrane protein 194A | 1.83 |
| 8050702 | TP53I3 | tumor protein p53 inducible protein 3 | 2.11 |
| 8140028 | TRIM50 | tripartite motif-containing 50 | 0.55 |
| 7964927 | TSPAN8 | tetraspanin 8 | 0.54 |
| 8176484 | TSPY2 | Testis specific protein | 0.54 |
| 8120838 | TTK | TTK protein kinase | 1.82 |
| 7990054 | UACA | uveal autoantigen with coiled-coil domains and ankyrin repeats | 1.92 |
| 8026490 | UCA1 | urothelial cancer associated 1 | 2.87 |
| 8095390 | UGT2B10 | UDP glucuronosyltransferase 2 family, polypeptide B10 | 0.55 |
| 8123388 | UNC93A | unc-93 homolog A (C. elegans) | 0.54 |
| 8040430 | VSNL1 | visinin-like 1 | 0.41 |
| 7938366 | WEE1 | WEE1 homolog (S. pombe) | 1.97 |
| 8038952 | ZNF616 | zinc finger protein 616 | 0.55 |
