# Supplementary material for: Integrated Proteomic and Transcriptomic Investigation of the Acetaminophen Toxicity in Liver Microfluidic Biochip
Source: PLoS One. 2011 Aug 8;6(8):e21268. doi: 10.1371/journal.pone.0021268 (PMC3152546; doi:10.1371/journal.pone.0021268)
Supplement: Table S2 — Differentially expressed genes by the APAP treatment in Petri when compared to the untreated Petri (fold change above 1.8). (DOC) [file pone.0021268.s002.doc]

**Supplementary table 2:** Differentially expressed genes by the APAP treatment in Petri when compared to the untreated Petri (fold change above 1.8).

| **Affymetrix** | **Gene Name** | **Complete Name** | **Fold Change** |
| --- | --- | --- | --- |
| 8154563 | ACER2 | alkaline ceramidase 2 | 2.15 |
| 7934906 | ACTA2 | actin, alpha 2, smooth muscle, aorta | 2.36 |
| 8132318 | ANLN | anillin, actin binding protein | 1.85 |
| 8059350 | AP1S3 | adaptor-related protein complex 1, sigma 3 subunit | 1.95 |
| 8073062 | APOBEC3B | apolipoprotein B mRNA editing enzyme, catalytic polypeptide-like 3B | 2.19 |
| 8160670 | AQP3 | aquaporin 3 (Gill blood group) | 2.04 |
| 8136987 | ARHGEF5 | other | 1.84 |
| 7986350 | ARRDC4 | arrestin domain containing 4 | 2.11 |
| 8034772 | ASF1B | ASF1 anti-silencing function 1 homolog B (S. cerevisiae) | 2.15 |
| 8006187 | ATAD5 | ATPase family, AAA domain containing 5 | 2.05 |
| 7986068 | BLM | Bloom syndrome | 2.26 |
| 8015769 | BRCA1 | breast cancer 1, early onset | 2.27 |
| 7968484 | BRCA2 | breast cancer 2, early onset | 1.83 |
| 8017262 | BRIP1 | BRCA1 interacting protein C-terminal helicase 1 | 2.12 |
| 7973948 | BRMS1L | breast cancer metastasis-suppressor 1-like | 1.84 |
| 7908917 | BTG2 | BTG family, member 2 | 2.63 |
| 7942832 | C11orf82 | chromosome 11 open reading frame 82 | 2.21 |
| 7953211 | C12orf5 | chromosome 12 open reading frame 5 | 1.88 |
| 8021286 | C18orf54 | chromosome 18 open reading frame 54 | 2.41 |
| 8124859 | C6orf15 | chromosome 6 open reading frame 15 | 0.34 |
| 8178439 | C6orf15 | chromosome 6 open reading frame 15 | 0.25 |
| 8121911 | C6orf173 | chromosome 6 open reading frame 173 | 2.02 |
| 7949227 | CDC42BPG | CDC42 binding protein kinase gamma (DMPK-like) | 1.81 |
| 8007071 | CDC6 | cell division cycle 6 homolog (S. cerevisiae) | 2.51 |
| 8012951 | CDRT1///TRIM16 | CMT1A duplicated region transcript 1 | 0.51 |
| 7997839 | CDT1 | chromatin licensing and DNA replication factor 1 | 1.80 |
| 8158976 | CEL | carboxyl ester lipase (bile salt-stimulated lipase) | 5.33 |
| 8168794 | CENPI | centromere protein I | 1.89 |
| 7997381 | CENPN | centromere protein N | 1.87 |
| 8068478 | CHAF1B | chromatin assembly factor 1, subunit B (p60) | 1.81 |
| 8002882 | CHST6 | carbohydrate (N-acetylglucosamine 6-O) sulfotransferase 6 | 2.24 |
| 8054702 | CKAP2L | cytoskeleton associated protein 2-like | 1.91 |
| 7914851 | CLSPN | claspin homolog (Xenopus laevis) | 1.95 |
| 8043657 | CNNM4 | cyclin M4 | 1.83 |
| 8082058 | CSTA | cystatin A (stefin A) | 1.87 |
| 8162652 | CTSL2 | cathepsin L2 | 1.93 |
| 8018754 | CYGB | cytoglobin | 2.65 |
| 8141342 | CYP3A7 | cytochrome P450, family 3, subfamily A, polypeptide 7 | 2.79 |
| 7958425 | DAO | D-amino-acid oxidase | 2.85 |
| 7939738 | DDB2 | damage-specific DNA binding protein 2, 48kDa | 2.27 |
| 7956046 | DGKA | diacylglycerol kinase, alpha 80kDa | 2.99 |
| 7958019 | DRAM1 | damage-regulated autophagy modulator | 1.92 |
| 8066074 | DSN1 | DSN1, MIND kinetochore complex component, homolog (S. cerevisiae) | 1.95 |
| 7909568 | DTL | denticleless homolog (Drosophila) | 2.08 |
| 7965094 | E2F7 | E2F transcription factor 7 | 3.46 |
| 8008310 | EME1 | essential meiotic endonuclease 1 homolog 1 (S. pombe) | 2.23 |
| 8173506 | ERCC6L | excision repair cross-complementing rodent repair deficiency, complementation group 6-like | 1.94 |
| 8145570 | ESCO2 | establishment of cohesion 1 homolog 2 (S. cerevisiae) | 2.05 |
| 7910997 | EXO1 | exonuclease 1 | 2.11 |
| 7948420 | FABP5L3///FABP5 | Putative fatty acid-binding protein 5-like protein 3 | 2.81 |
| 8052554 | FAM161A | family with sequence similarity 161, member A | 1.85 |
| 8136115 | FAM40B | family with sequence similarity 40, member B | 1.95 |
| 8077731 | FANCD2 | Fanconi anemia, complementation group D2 | 1.86 |
| 7985829 | FANCI | Fanconi anemia, complementation group I | 1.85 |
| 8018236 | FDXR | ferredoxin reductase | 2.36 |
| 8139632 | FIGNL1 | fidgetin-like 1 | 1.93 |
| 7933446 | FRMPD2 | FERM and PDZ domain containing 2 | 1.95 |
| 8141459 | GAL3ST4 | galactose-3-O-sulfotransferase 4 | 1.82 |
| 8040440 | GEN1 | Gen homolog 1, endonuclease (Drosophila) | 1.84 |
| 8061471 | GINS1 | GINS complex subunit 1 (Psf1 homolog) | 1.92 |
| 8165663 | GPAM | Glycerol 3 phosphate acetyl transferase 1 | 0.42 |
| 7996081 | GPR56 | G protein-coupled receptor 56 | 1.82 |
| 8124394 | HIST1H2BB | histone cluster 1, H2bb | 0.49 |
| 8117594 | HIST1H2BM | histone cluster 1, H2bm | 0.39 |
| 8178220 | HLA-DPB1 | major histocompatibility complex, class II, DP beta 1 | 0.55 |
| 7997188 | HP |  | 0.50 |
| 8097335 | HSPA4L | heat shock 70kDa protein 4-like | 1.93 |
| 7981722 | IGHA1///IGHG1 | immunoglobulin heavy constant alpha 1 | 0.35 |
| 7956426 | INHBE | inhibin, beta E | 0.49 |
| 7921033 | IQGAP3 | IQ motif containing GTPase activating protein 3 | 1.82 |
| 8035465 | ISYNA1 | inositol-3-phosphate synthase 1 | 1.85 |
| 8078227 | KAT2B | K(lysine) acetyltransferase 2B | 2.04 |
| 8031346 | KIR2DL4///KIR2DL5A///KIR2DL3 | killer cell immunoglobulin-like receptor | 0.50 |
| 8020551 | LAMA3 | laminin, alpha 3 | 1.84 |
| 7914270 | LAPTM5 | Homo sapiens lysosomal protein transmembrane 5 | 2.40 |
| 7929373 | LGI1 | leucine-rich, glioma inactivated 1 | 1.83 |
| 7914878 | LOC100289612 | Protein coding | 2.05 |
| 7926821 | MASTL | microtubule associated serine/threonine kinase-like | 2.00 |
| 7926259 | MCM10 | minichromosome maintenance complex component 10 | 1.94 |
| 8060813 | MCM8 | minichromosome maintenance complex component 8 | 1.88 |
| 8155214 | MELK | maternal embryonic leucine zipper kinase | 1.82 |
| 7955441 | METTL7A | methyltransferase like 7A | 1.84 |
| 8177955 | MICB | MHC class I polypeptide-related sequence B | 1.93 |
| 8174313 | MORC4 | MORC family CW-type zinc finger 4 | 2.10 |
| 8174322 | MORC4 | MORC family CW-type zinc finger 4 | 1.84 |
| 7907893 | MR1 | major histocompatibility complex, class I-related | 2.74 |
| 8090180 | MUC13 | mucin 13, cell surface associated | 3.08 |
| 7903478 | NBPF6///NBPF4///NBPF5 | neuroblastoma breakpoint family, member 6 | 3.03 |
| 8133314 | NCF1///NCF1C///NCF1B | Neutrophil cytosolic factor 1 | 2.49 |
| 8098423 | NEIL3 | nei endonuclease VIII-like 3 (E. coli) | 1.89 |
| 8165217 | NOTCH1 | Notch homolog 1, translocation-associated (Drosophila) | 2.06 |
| 7957835 | NR1H4 | nuclear receptor subfamily 1, group H, member 4 | 0.43 |
| 8157446 | ORM1 | orosomucoid 1 | 0.53 |
| 7961026 | OVOS2///OVOS///LOC728715 | Ovostatin homolog 2 precursor | 1.93 |
| 8163672 | PAPPA///PAPPAS | pregnancy-associated plasma protein A, pappalysin 1 | 1.89 |
| 8064844 | PCNA | proliferating cell nuclear antigen | 1.85 |
| 8057959 | PGAP1 | post-GPI attachment to proteins 1 | 1.89 |
| 7901054 | PLK3 | polo-like kinase 3 (Drosophila) | 1.84 |
| 8066619 | PLTP | phospholipid transfer protein | 2.68 |
| 7941214 | POLA2 | polymerase (DNA directed), alpha 2 (70kD subunit) | 2.22 |
| 8119858 | POLH | polymerase (DNA directed), eta | 2.10 |
| 8089875 | POLQ | polymerase (DNA directed), theta | 2.14 |
| 8074714 | POM121L9P | membrane glycoprotein-like 9 | 0.31 |
| 8008922 | PPM1D | protein phosphatase 1D magnesium-dependent, delta isoform | 1.85 |
| 8176806 | PRY///PRY2 | PTPN13-like, Y-linked 2 | 1.82 |
| 8023043 | PSTPIP2 | proline-serine-threonine phosphatase interacting protein 2 | 3.49 |
| 7899562 | PTPRU | protein tyrosine phosphatase, receptor type, U | 2.37 |
| 7921773 | PVRL4 | poliovirus receptor-related 4 | 2.07 |
| 8044669 | RABL2A///RABL2B | member of RAS oncogene family-like 2A//2B | 3.05 |
| 7982792 | RAD51 | RAD51 homolog (RecA homolog, E. coli) (S. cerevisiae) | 2.15 |
| 8008754 | RAD51C | RAD51 homolog C (S. cerevisiae) | 1.96 |
| 8108359 | REEP2 | receptor accessory protein 2 | 2.14 |
| 8002762 | RFWD3 | ring finger and WD repeat domain 3 | 1.85 |
| 8142079 | RINT1///EFCAB10 | EF-hand calcium binding domain 10 | 2.33 |
| 8152133 | RRM2B | ribonucleotide reductase M2 B (TP53 inducible) | 1.82 |
| 8079896 | SEMA3F | sema domain, immunoglobulin domain (Ig), short basic domain, secreted, (semaphorin) 3F | 1.93 |
| 8085754 | SGOL1 | shugoshin-like 1 (S. pombe) | 1.91 |
| 8001133 | SHCBP1 | SHC SH2-domain binding protein 1 | 1.96 |
| 7970513 | SKA3 | chromosome 13 open reading frame 3 | 1.99 |
| 8064613 | SLC4A11 | solute carrier family 4, sodium borate transporter, member 11 | 1.94 |
| 8068361 | SLC5A3 | solute carrier family 5 (sodium/myo-inositol cotransporter), member 3 | 3.06 |
| 8068353 | SLC5A3///MRPS6 | solute carrier family 5 | 1.82 |
| 8102800 | SLC7A11 | solute carrier family 7, (cationic amino acid transporter, y+ system) member 11 | 0.41 |
| 8095021 | SPATA18 | spermatogenesis associated 18 homolog (rat) | 1.93 |
| 7914180 | SPCS2///LOC653566 | signal peptidase complex subunit 2 | 1.88 |
| 8133654 | SPDYE1 | speedy homolog E1 | 2.23 |
| 8096301 | SPP1 | secreted phosphoprotein 1 | 0.53 |
| 8066822 | SULF2 | sulfatase 2 | 3.05 |
| 8045889 | TANC1 | tetratricopeptide repeat, ankyrin repeat and coiled-coil containing 1 | 1.84 |
| 8180061 | TAP1 | transporter 1, ATP-binding cassette, sub-family B (MDR/TAP) | 2.24 |
| 8177947 | TCF19 | transcription factor 19 | 2.73 |
| 7900658 | TIE1 | tyrosine kinase with immunoglobulin-like and EGF-like domains 1 | 1.98 |
| 8018849 | TK1 | thymidine kinase 1, soluble | 2.36 |
| 7961983 | TM7SF3 | transmembrane 7 superfamily member 3 | 1.81 |
| 7964347 | TMEM194A | transmembrane protein 194A | 2.02 |
| 8171472 | TMEM27 | transmembrane protein 27 | 2.28 |
| 8095364 | TMPRSS11E | transmembrane protease, serine 11E | 1.83 |
| 7937749 | TNNT3 | troponin T type 3 (skeletal, fast) | 1.95 |
| 8050702 | TP53I3 | tumor protein p53 inducible protein 3 | 3.65 |
| 8151890 | TP53INP1 | tumor protein p53 inducible nuclear protein 1 | 2.64 |
| 7938035 | TRIM22///OR56B1 | tripartite motif-containing 22 | 2.23 |
| 7964927 | TSPAN8 | tetraspanin 8 | 0.43 |
| 8176484 | TSPY2 | Testis specific protein | 1.81 |
| 8062844 | TTPAL | tocopherol (alpha) transfer protein-like | 1.83 |
| 7904726 | TXNIP | thioredoxin interacting protein | 2.79 |
| 8026490 | UCA1 | urothelial cancer associated 1 | 2.66 |
| 7952249 | USP2 | ubiquitin specific peptidase 2 | 1.96 |
| 7979281 | WDHD1 | WD repeat and HMG-box DNA binding protein 1 | 2.02 |
| 8144036 | XRCC2 | X-ray repair complementing defective repair in Chinese hamster cells 2 | 1.88 |
| 8035838 | ZNF724P | Putative zinc finger protein 724 | 2.17 |
| 8031799 | ZSCAN4 | zinc finger and SCAN domain containing 4 | 1.96 |
