# Supplementary material for: Integrated Proteomic and Transcriptomic Investigation of the Acetaminophen Toxicity in Liver Microfluidic Biochip
Source: PLoS One. 2011 Aug 8;6(8):e21268. doi: 10.1371/journal.pone.0021268 (PMC3152546; doi:10.1371/journal.pone.0021268)
Supplement: Table S3 — Proteins differentially expressed by the biochip versus the Petri and successfully identified by MS/MS. (DOC) [file pone.0021268.s003.doc]

**Supplementary table 3**: Proteins differentially expressed by the biochip versus the Petri and successfully identified by MS/MS

| **Identifiant swissprot** | **Gene Name** | **Complete Name** | **Accession number swiss prot** | **Fold change** |
| --- | --- | --- | --- | --- |
| ACDSB_HUMAN | ACADSB | Short/branched chain specific acyl-CoA dehydrogenase, mitochondrial | P45954 | -1.8 |
| THIC_HUMAN | ACAT1 | Acetyl-CoA acetyltransferase | Q9BWD1 | 1.5 |
| ATCG_HUMAN | ACTG1 | Actine, cytoplasmique | P63261 | 1.7 |
| FETA_HUMAN | AFP | Alpha-fetoprotein | P02771 | 3.4 |
| ALBU_HUMAN | ALB | Serum albumin | P02768 | 2.3 |
| AL1A1_HUMAN | ALDH1A1 | Retinal dehydrogenase 1 | P00352 | 2 |
| AL1B1_HUMAN | ALDH1B1 | Aldehyde dehydrogenase X mitochondrial | P30837 | 1.4 |
| AMGO2_HUMAN | AMIGO2 | Amphoterin-induced protein 2 | Q86SJ2 | -1.8 |
| ANXA6_HUMAN | ANXA6 | Anexin A6 | P08133 | 1.5 |
| ANXA7_HUMAN | ANXA7 | Annexin A7 | P20073 | -2.6 |
| KCRB_HUMAN | CKB | Creatine kinase B | P12277 | -2.7 |
| COR1B_HUMAN | CORO1B | Coronin-1B | Q9BR76 | -1.8 |
| CATB_HUMAN | CTSB | Cathepsine B | P07858 | 2 |
| CATB_HUMAN | CTSB | Cathepsine B | P07858 | -2.4* |
| CATC_HUMAN | CTSC | Dipeptidyl-peptidase 1 | P53664 | -1.4 |
| CATD_HUMAN | CTSD | Cathepsine D | P07339 | -2.1 |
| P07339 | CTSD | Cathepsin D | P07339 | -1.6 |
| SRC8_HUMAN | CTTN | Src substrate cortactin | Q14247 | -1.7 |
| DD19B_HUMAN | DDX19B | ATP-dependent RNA helicaseDDX19B | Q9UMR2 | 2.7 |
| ODP2_HUMAN | DLAT | Dihydrolipoyllysine-residue acetyltransferase component of pyruvate dehydrogenase complex | P10515 | 1.8 |
| DPP4_HUMAN | DPP4 | Dipeptidyl peptidase 4 | P27487 | -1.9 |
| E2F8_HUMAN | E2F8 | Transcription factor E2F8 | A0AVK6 | 1.9 |
| EF2_HUMAN | EEF2 | Elongation Factor 2 | P13639 | 1.5 |
| EIF3F_HUMAN | EIF3F | Eukaryotic translation initiation factor 3 subunit F | O00303 | -2.1 |
| EiF5A1 | **EIF5A** | Eukaryotic translation initiation factor 5A-1 | P63241 | 1.5 |
| ENOA_HUMAN | ENO1 | Alpha-enolase | P06733 | -1.7 |
| ENOG_HUMAN | ENO2 | Gamma-enolase | P09104 | -2.1 |
| ERO1A_HUMAN | ERO1L | ERO1-like protein alpha | Q96HE7 | -2.1 |
| ERP29_HUMAN | ERP29 | Endoplasmic reticulum resident protein 29 | P30040 | 1.6 |
| FRIL_HUMAN | FTL | (Ferritin light chain) | P02792 | 1.7 |
|  | GALM | Aldose 1 epimérase | Q96C23 | 1.5 |
| GRPL2_HUMAN | GLIPR1L2 | GLIPR1-like protein2 | Q4G1C9 | -1.6 |
| GLSK_HUMAN | GLS | Glutaminase kidney isoform, mitochondrial | O94925 | 2.7 |
| GRPE1 | GRPEL1 | GrpE protein homolog 1 | Q9HAV7 | 1.9 |
| GRSF1_HUMAN | GRSF1 | G-rich sequence factor 1 | Q12849 | 1.6 |
| GSTO1 | GSTO1 | Glutathion transférase omega 1 | P78417 | 2.4 |
| HMCS1_HUMAN | HMGCS1 | Hydroxymethylglutaryl-CoA synthase, cytoplasmic | Q01581 | 2.5 |
| HMCS1_HUMAN | HMGCS1 | Hydroxymethylglutaryl-CoA synthetase | Q01581 | -1.4 |
| HNRPC_HUMAN | HNRPC | Heterogeneous nuclear ribonucleoproteins C1/C2 | P07910 | 1.9* |
| HS90A_HUMAN | HSP90AA1 | Heat shock protein HSP 90-alpha | P07900 | 1.6 |
| HS90B_HUMAN | HSP90AB1 | Heat shock protein HSP 90-beta | P08238 | 1.6 |
| ENPL_HUMAN | HSP90B1 | Endoplasmin | P14625 | -1.8 |
| GRP78_HUMAN | HSPA5 | 78 kDa glucose-regulated protein | P11021 | -1.7 |
| HSP7C_HUMAN | HSPA8 | Heat shock cognate 71 kDa protein | P11142 | 1.6 |
| IDE_HUMAN | IDE | Insulin-degrading enzyme | P14735 | 1.8 |
| K2C1_HUMAN | KRT1 | Keratin, type II cytoskeletal 1 | P04264 | 1.5 |
| K2C1_HUMAN | KRT1 | Keratin, type II cytoskeletal 1 | P04264 | -1.8 |
| K1C18_HUMAN | KRT18 | Keratin, type I cytoskeletal 18 | P05783 | -1.6 |
| K2C8_HUMAN | KRT8 | Keratin, type II cytoskeletal 8 | P05787 | -2.1 |
| LACB2_HUMAN | LACTB2 | Beta-lactamase-like protein 2 | Q53H82 | -1.8 |
| LEG12_HUMAN | LGALS12 | Galectin-12 | Q96DT0 | -1.6 |
| MCM7_HUMAN | MCM7 | DNA replication licensing factor MCM7 | P33993 | 1.6 |
| RM12_HUMAN | **MRPL12** | 39S ribosomal protein L12 | P52815 | 1.8 |
| NACA_HUMAN | NACA | Nascent polypeptide-associated complex subunit alpha | Q13765 | 2.5 |
| NUCL_HUMAN | NCL | Nucleolin | P19338 | 1.7 |
| NDRG1_HUMAN | NDRG1 | Protein NDRG1 | Q92597 | -4.3 |
| NDUS1_HUMAN | NDUFS1 | NADH-ubiquinone oxidoreductase 75 kDa subunit | P28331 | -1.6 |
| NT5C_HUMAN | NT5C | 5'(3')-deoxyribonucleotidase, cytosolic type | Q8TCD5 | -2.1 |
| PAIP1_HUMAN | PAIP1 | Polyadenylate-binding protein-interacting protein 1 | Q9H074 | 1.6 |
| PARK7 | PARK7 | Protéine DJ-1 | Q99497 | 2 |
| PCNA_HUMAN | PCNA | Proliferating cell nuclear antigen | P12004 | 2.3 |
| PDIA3_HUMAN | PDIA3 | Protein disulfide-isomerase A3 | P30101 | -1.5 |
| PHB | PHB | Prohibitin | P352 32 | 2.4 |
| SERA_HUMAN | PHGDH | D-3-phosphoglycerate dehydrogenase | O43175 | 2.1 |
| M6PBP_HUMAN | PLIN3 | Mannose-6-phosphate receptor-binding protein 1 | O60664 | -2.1 |
| POTEE_HUMAN | POTEE | POTE ankyrin domain family member E | Q6S8J3 | 1.7 |
| RBBP4_HUMAN | RBBP4 | Histone-binding protein RBBP4 | Q09028 | 1.6 |
| RCN2_HUMAN | RCN2 | Reticulocalbin-2 | Q14257 | 2 |
| RING2_HUMAN | RNF2 | E3 ubiquitin-protein ligase RING2 | Q99496 | -1.5 |
| BRE1A_HUMAN | RNF20 | E3 ubiquitin-protein ligase BRE1A | Q5VTR2 | -1.6 |
| S100P_HUMAN | S100P | Protein S100-P | P25815 | -2.2 |
| DHSA_HUMAN | SDHA | Succinate dehydrogenase [ubiquinone] flavoprotein subunit | P31040 | -2 |
| SEP14_HUMAN | SEPT14 | Septin-14 | Q6ZU15 | -1.5 |
| SPB8_HUMAN | Serpin B8 | Serpin B8 | P50452 | -1.9 |
| A1AT_HUMAN | SERPINA1 | Alpha-1-antitrypsin | P0109 | 3.5 |
| ILEU_HUMAN | SERPINB1 | Leukocyte elastase inhibitor | P30740 | -1.9 |
| 1433S_HUMAN | SFN | 14-3-3 protein sigma | P31947 | 1.5 |
| SPRC_HUMAN | SPARC | SPARC | P09486 | -2.1 |
| SPTA2_HUMAN | SPTAN1 | Spectrin alpha chain, Brain | Q13813 | -1.8 |
| ST1A1_HUMAN | ST1A1 | Sulfotransférase 1A1 | P50225 | 1.5 |
| STRAP_HUMAN | STRAP | Serine-threonine kinase receptor-associated protein | Q9Y3F4 | 1.4 |
| TALDO_HUMAN | TALDO1 | Transaldolase | P37837 | 1.5 |
| TIM50_HUMAN | TIMM50 | Mitochondrial import inner membrane translocase subunit TIM50 | Q3ZCQ8 | 1.6 |
| TPIS_HUMAN | TPI1 | Triosephosphate isomerase | P60174 | -1.9 |
| TBA1B_HUMAN | TUBA1B | Tubulin alpha-1B chain | P68363 | -1.6 |
| TRXR1_HUMAN | TXNRD1 | Thioredoxin reductase 1, cytoplasmic | Q16881 | 1.9/3.1 |
| VAT1_HUMAN | VAT1 | Synaptic vesicle membrane protein VAT-1 homolog | Q99536 | 1.6 |
| VISL1_HUMAN | VSNL1 | Visinin-like protein 1 | P62760 | -1.5 |
| 1433G_HUMAN | YWHAG | 14-3-3 protein gamma | P61981 | 1.8 |
