# Supplementary material for: Integrated Proteomic and Transcriptomic Investigation of the Acetaminophen Toxicity in Liver Microfluidic Biochip
Source: PLoS One. 2011 Aug 8;6(8):e21268. doi: 10.1371/journal.pone.0021268 (PMC3152546; doi:10.1371/journal.pone.0021268)
Supplement: Table S4 — Differentially expressed proteins by the treatment effect in biochip and successfully identified by MS/MS. (DOC) [file pone.0021268.s004.doc]

**Supplementary table 4:** Differentially expressed proteins by the treatment effect in biochip and successfully identified by MS/MS

| **Identifiant**  **swissprot** | **Gène** | **Complete Name** | **Accession number swiss prot** | **Fold change** |
| --- | --- | --- | --- | --- |
| CATB_HUMAN | CTSB | Cathepsine B | P07858 | 1.5 |
| CASP3 | CASP3 | Caspase 3 | P42574 | 1.5 |
| ADFP_HUMAN | PLIN2 | Adipophilin | Q99541 | 1.7 |
| G6PD_HUMAN | G6PD | Glucose-6-phosphate 1-dehydrogenase | P11413 | 1.5 |
| TRXR1_HUMAN* | TXNRD1 | Thioredoxin reductase 1, cytoplasmic | Q16881 | 1.8 |
| K2C8_HUMAN | KRT8 | Keratin, type II cytoskeletal 8 | P05787 | 1.7 |
| SEP14_HUMAN | SEPT14 | Septin-14 | Q6ZU15 | 1.6 |
| RING2_HUMAN | RNF2 | E3 ubiquitin-protein ligase RING2 | Q99496 | 2.1 |
| BRE1A_HUMAN | RNF20 | E3 ubiquitin-protein ligase BRE1A | Q5VTR2 | 2.1 |
| DPP4_HUMAN | DPP4 | Dipeptidyl peptidase 4 | P27487 | 1.5 |
| SRC8_HUMAN | CTTN | Src substrate cortactin | Q14247 | 1.8 |
| DPYL2_HUMAN | DPYSL2 | Dihydropyrimidinase-related protein 2 | Q16555 | 1.7 |
| LKHA4_HUMAN | LTA4H | Leukotriene A-4 hydrolase | P09960 | 1.7 |
| PP2AA_HUMAN | PPP2CA | Serine/threonine-protein phosphatase 2A catalytic subunit alpha isoform | P67775 | 1.8 |
| S100P_HUMAN | S100P | Protein S100-P | P25815 | -1.6 |
| TBA1C_HUMAN | TUBA1C | Tubulin alpha-1C chain | Q9BQE3 | -2.3 |
| KRT86_HUMAN | KRT86 | Keratin, type II cuticular Hb6 | O43790 | -1.6 |
| ROAA_HUMAN | HNRNPAB | Heterogeneous nuclear ribonucleoprotein A/B | Q99729 | -1.6 |
| ATCG_HUMAN | ACTG1 | Actin, cytoplasmic | P63261 | -1.8 |
| POTEE_HUMAN | POTEE | POTE ankyrin domain family member E | Q6S8J3 | -1.8 |
| HNRPC_HUMAN | HNRPC | Heterogeneous nuclear ribonucleoproteins C1/C2 | P07910 | -1.5 |
| STRAP_HUMAN | STRAP | Serine-threonine kinase receptor-associated protein | Q9Y3F4 | -1.5 |
| FETA_HUMAN | AFP | Alpha-fetoprotein | P02771 | -1.8 |
| COR1B_HUMAN | CORO1B | Coronin-1B | Q9BR76 | -1.8 |
| K2C1_HUMAN | KRT1 | Keratin, type II cytoskeletal 1 | P04264 | -1.8 |
| AMGO2_HUMAN | AMIGO2 | Amphoterin-induced protein 2 | Q86SJ2 | -1.8 |
| SPTA2_HUMAN | SPTAN1 | Spectrin alpha chain, Brain | Q13813 | -1.8 |
| SRC8_HUMAN | CTTN | Src substrate cortactin | Q14247 | -1.7 |
| K2C8_HUMAN | KRT8 | Keratin, type II cytoskeletal 8 | P05787 | -1.5 |
| ANXA7_HUMAN | ANXA7 | Annexin A7 | P20073 | -1.4 |
| VISL1_HUMAN | VSNL1 | Visinin-like protein 1 | P62760 | -1.4 |
