# Supplementary material for: Integrated Proteomic and Transcriptomic Investigation of the Acetaminophen Toxicity in Liver Microfluidic Biochip
Source: PLoS One. 2011 Aug 8;6(8):e21268. doi: 10.1371/journal.pone.0021268 (PMC3152546; doi:10.1371/journal.pone.0021268)
Supplement: Table S5 — Differentially expressed proteins by the treatment effect in Petri dishes and successfully identified by MS/MS. (DOC) [file pone.0021268.s005.doc]

**Supplementary table 5:** Differentially expressed proteins by the treatment effect in Petri dishes and successfully identified by MS/MS

| **Identifiant**  **Swissprot** | **Gene Name** | **Complete Name** | **Accession number**  **swissprot** | **Fold change** |
| --- | --- | --- | --- | --- |
| ANXA7_HUMAN | ANXA7 | Annexin A7 | P20073 | -2.1 |
| CAP2_HUMAN | CAP2 | Adenylyl cyclase-associated protein2 | P40123 | -1.6 |
| COR1B_HUMAN | CORO1B | Coronin-1B | Q9BR76 | -1.8 |
| SRC8_HUMAN | CTTN | Src substrate cortactin | Q14247 | -1.7 |
| EIF3F_HUMAN | EIF3F | Eukaryotic translation initiation factor 3 subunit F | O00303 | 1.6 |
| ROAA_HUMAN | HNRNPAB | Heterogeneous nuclear ribonucleoprotein A/B | Q99729 | 2.1 |
| KIF6_HUMAN | KIF6 | Kinesin-like protein KIF6 | Q6ZMV9 | -1.6 |
| K2C8_HUMAN | KRT8 | Keratin, type II cytoskeletal 8 | P05787 | 1.7 |
| KRT86_HUMAN | KRT86 | Keratin, type II cuticular Hb6 | O43790 | 2.1 |
| NUCL_HUMAN | NCL | Nucleolin | P19338 | 1.6 |
| PCNA_HUMAN | PCNA | Proliferating cell nuclear antigen | P12004 | 1.5 |
| M6PBP_HUMAN | PLIN3 | Mannose-6-phosphate receptor-binding protein 1 | O60664 | 1.6 |
| A1AT_HUMAN | SERPINA1 | Alpha-1-antitrypsin | P0109 | 1.9 |
| TBA1C_HUMAN | TUBA1C | Tubulin alpha-1C chain | Q9BQE3 | -2.3 |
| MCM7_HUMAN | MCM7 | DNA replication licensing factor MCM7 | P33993 | 1.5 |
| RBBP4_HUMAN | RBBP4 | Histone-binding protein RBBP4 | Q09028 | 1.4 |
