# Supplementary material for: Integrated Proteomic and Transcriptomic Investigation of the Acetaminophen Toxicity in Liver Microfluidic Biochip
Source: PLoS One. 2011 Aug 8;6(8):e21268. doi: 10.1371/journal.pone.0021268 (PMC3152546; doi:10.1371/journal.pone.0021268)
Supplement: Table S6 — Experimental design and samples labelling of the proteomic analysis. Each biochip and Petri is the pool of three independent replicates. (DOC) [file pone.0021268.s006.doc]

**Supplementary Table 6:** Experimental design and samples labelling of the proteomic analysis. Each biochip and Petri is the pool of three independent replicates

| **Gel number** | **Cy2 dye** | **Cy3 dye** | **Cy5 dye** |
| --- | --- | --- | --- |
| 1 | Internal Standard | Biochip Control 1 | Petri control 3 |
| 2 | Internal Standard | Biochip Control 2 | Petri treated 3 |
| 3 | Internal Standard | Petri Control 1 | Biochip treated 3 |
| 4 | Internal Standard | Petri Control 2 | Petri traited 4 |
| 5 | Internal Standard | Petri treated 1 | Biochip control 3 |
| 6 | Internal Standard | Petri treated 2 | Biochip treated 4 |
| 7 | Internal Standard | Biochip treated 1 | Biochip control 4 |
| 8 | Internal Standard | Biochip treated 2 | Petri control 4 |
